# Comparative evaluation of clinical and cerebrospinal fluid biomarker characteristics in rapidly and non-rapidly progressive Alzheimer’s disease

**DOI:** 10.1186/s13195-023-01249-y

**Published:** 2023-06-08

**Authors:** Janne Marieke Herden, Peter Hermann, Isabel Schmidt, Kathrin Dittmar, Sezgi Canaslan, Luise Weglage, Sabine Nuhn, Corinna Volpers, Astrid Schlung, Stefan Goebel, Fabian Kück, Anna Villar-Piqué, Christian Schmidt, Dirk Wedekind, Inga Zerr

**Affiliations:** 1 Department of Neurology, Clinical Dementia Center and National Reference Center for CJD Surveillance, University Medical Center, Robert-Koch-Straße 40, Göttingen, 37075 Germany; 2 German Center for Neurodegenerative Diseases (DZNE), Göttingen, Germany; 3 Department of Medical Statistics, University Medical Center Göttingen, Humboldtallee 32, Göttingen, 37073 Germany; 4 Neurologische Gemeinschaftspraxis Am Groner Tor, Göttingen, Germany; 5 Department of Psychiatry and Psychotherapy, University Medical Center, Von-Siebold-Straße 5, Göttingen, 37075 Germany

**Keywords:** Alzheimer’s disease, Rapidly progressive Alzheimer’s disease, Phenotype, Biomarkers, APOE

## Abstract

**Background:**

Rapidly progressive forms of Alzheimer’s disease (rpAD) are increasingly recognized and may have a prevalence of up to 30% of patients among all patients with Alzheimer’s disease (AD). However, insights about risk factors, underlying pathophysiological processes, and clinical characteristics of rpAD remain controversial. This study aimed to gain a comprehensive picture of rpAD and new insights into the clinical manifestation to enable a better interpretation of disease courses in clinical practice as well as in future clinical studies.

**Methods:**

Patients (*n* = 228) from a prospective observational study on AD were selected and categorized into rpAD (*n* = 67) and non-rpAD (*n* = 161) disease groups. Patients were recruited through the German Creutzfeldt-Jakob disease surveillance center and the memory outpatient clinic of the Göttingen University Medical Center, representing diverse phenotypes of the AD population. Biomarkers and clinical presentation were assessed using standardized protocols. A drop of ≥ MMSE 6 points within 12 months defined rapid progressors.

**Results:**

Lower CSF Amyloid beta 1–42 concentrations (*p* = 0.048), lower Amyloid beta 42/40 ratio (*p* = 0.038), and higher Tau/Amyloid-beta 1–42 ratio, as well as pTau/Amyloid-beta 1–42 ratio (each *p* = 0.004) were associated with rpAD. Analyzes in a subset of the cohort (rpAD: *n* = 12; non-rpAD: *n* = 31) showed higher CSF NfL levels in rpAD (*p* = 0.024). Clinically, rpAD showed earlier impairment of functional abilities (*p* < 0.001) and higher scores on the Unified Parkinson’s Disease Rating Scale III (*p* < 0.001), indicating pronounced extrapyramidal motor symptoms. Furthermore, cognitive profiles (adjusted for overall cognitive performance) indicated marked deficits in semantic (*p* = 0.008) and phonematic (0.023) verbal fluency tests as well as word list learning (*p* = 0.007) in rpAD compared to non-rpAD. The distribution of APOE genotypes did not differ significantly between groups.

**Conclusions:**

Our results suggest that rpAD is associated with distinct cognitive profiles, earlier occurrence of non-cognitive symptoms, extrapyramidal motoric disturbance, and lower Amyloid-beta 1–42 concentrations in the CSF. The findings may help to characterize a distinct phenotype of rpAD and estimate prognosis based on clinical characteristics and biomarker results. However, an important future goal should be a unified definition for rpAD to enable targeted study designs and better comparability of the results.

**Supplementary Information:**

The online version contains supplementary material available at 10.1186/s13195-023-01249-y.

## Background

Alzheimer’s disease (AD) is the most common neurodegenerative dementia worldwide, with increasing incidence [1]. The course and progression of the disease are variable and early and late onset; limbic and cortical type and atypical forms have been described. Several studies have suggested a rapidly progressive subtype [2, 3], and the presence of such a subtype may pose severe problems for research as it can significantly complicate the interpretation of results of clinical studies [4]. The development of a disease-modifying therapy in the near future may be the most important goal in dementia research [5]. Besides improved estimation of prognosis, a better understanding of individual AD subtypes especially rapid disease progression is essential to design clinical trials more individually and to enable interpretation.

Depending on the underlying definition, up to 30% of patients with AD appear to have a rapid course [4], which illustrates that this subtype of AD is not a rare phenomenon. A wide variety of factors have been associated with rpAD in the literature. The underlying pathology of rapid cognitive decline in AD has not been clarified but was associated with the presence of amyloid angiopathy [6], distinct protein or aggregate structures of Amyloid-beta [7, 8], or factors that modulate Tau pathology [9]. Regarding demographic data, several studies associated earlier age of onset [10–13], higher educational attainment [10, 13], early focal neurological signs, and involvement of the extrapyramidal system [10, 13, 14] with faster cognitive decline. In clinical terms, rpAD may be associated with the earlier and increased onset of Behavioral and Psychological Symptoms of Dementia (BPSD) [14]. In addition, rpAD may also present specific biomarker profiles. For example, lower CSF Amyloid-beta 1–42 (Aβ1-42) concentrations [15] and a lower Aβ-ratio [16] and high Tau/Aβ1-42 ratio could be related to faster cognitive and functional deterioration [15, 17]. In addition, there is a presumption that the Tau levels in rpAD patients may be higher than those in “classical” AD (non-rpAD) patients [3, 4]. The apolipoprotein E4 allele (APOE4) is considered the most important genetic risk factor for sporadic AD [13, 17] and its role in the progression of cognitive and possibly functional impairment remains controversial [17, 18].

This study aimed to identify and characterize distinct clinical and laboratory profiles in patients with non-rpAD and rpAD, which were differentiated by the rate of annual drop MMSE scores [4]. This may help to gain a more comprehensive picture of the clinical manifestation of rpAD and to develop a valid definition of the disease subtype to enable a better interpretation of clinical studies in the future.

## Methods

### Study design, participants, and data acquisition

The unicentric longitudinal rpAD study was launched in 2008 and recruited patients from the Dementia Outpatient Clinic of the University Medical Centre Göttingen and the German National CJD Surveillance Unit between 2008 and 2021. As part of the study, patients were followed up for several years with annual examinations.

A total of 228 patients were selected for this study. All patients met the following inclusion criteria:

1. Patients fulfilled clinical diagnostic criteria [19, 20]
2. To increase diagnostic accuracy, at least one biomarker criterion from the A/T/N system [21] was AD-typical (amyloid in CSF or PET, p-Tau in CSF, temporo-medial atrophy in MRI)
3. Patients had at least one Mini-Mental State Examination (MMSE) result in addition to the one at baseline at an interval of no more than 12 months, the classification of disease progression into rpAD and non-rpAD.

All patients received a detailed medical history, clinical examination, and neuropsychological tests (MMSE [22] in all patients and CERAD-Plus [23] test battery in *n* = 56 non-rpAD and *n* = 41 rpAD patients). In addition, a variety of clinical scores such as Unified Parkinson’s Disease Rating Scale-III [24] (UPDRSIII, 0–108 pts), basic-Activity of Daily Living (ADL) [25] (BADL, 0–48 pts), instrumental-ADL [26] (IADL, 0–8 pts), and modified depression scale [27] (0–10 pts), were collected. Furthermore, structured interviews with relatives were performed.

### Group definition

Within the scope of the work, a group comparison was made between the rapidly and non-rapid progressive forms of Alzheimer’s disease. For this purpose, the definitions of rapidly progressive Alzheimer’s dementia by Soto et al. 2008 [28] and Schmidt et al. 2011 [4] were used. All patients in the rpAD group showed a drop of ≥ 6 points on the MMSE score within 12 months or less; all slow progressors (non-rpAD) lost less than 6 points within 12 months of observation.

### Evaluation of clinical symptoms and biomarker analyzes

To compare clinical characteristics, the two groups were analyzed for the presence of focal neurological symptoms and behavioral and psychiatric symptoms. This analysis is based on symptom complexes comprising a range of individual symptoms. The symptom complexes were only differentiated by presence and absence. The presence of one sign was sufficient.

Biomarker assessment for this study included the cerebrospinal fluid (CSF) proteins Amyloid-beta and Tau. Quantifying Tau, phosphorylated Tau protein (p-Tau), Aβ1-40, and Aβ1-42 was done using the ELISA kits (Fujirebio, Ghent Belgium). Due to internal changes in laboratory methods since 2010, the values for Aβ1-40 had to be normalized (see Additional file 1 A). In addition to CSF parameters, the ApoE genotype of the study participants was also included in the analyses. The ApoE genotype was determined with the help of DNA strip technology (Hain Lifescience).

A panel of additional CSF biomarkers had been analyzed in a subset of the cohort for previous studies and was here re-evaluated in the context of rpAD with consideration of disease stage as a potential influencing factor: NfL [29], total Prion-Protein (t-PrP) [30], chitinase-3-like protein 1 (YKL-40) [31], lipocalin 2 (LCN2) [32], and α-synuclein [33].

### Statistical methods

Statistics were performed using *GraphPad* Prism software (version 9.3.1). Sex, ApoE-genotypes, and clinical symptoms of the rpAD and the non-rpAD-group were compared using *Fisher’s exact test*. The Mann–Whitney *U* test was used to compare age, education, MMSE, CSF parameters, UPDRS III, ADL scores, and depression scale. The significance level was set at *p* ≤ 0.05. Subsequently, the group comparisons were corrected for age by a multiple logistic regression model. Moreover, disease duration at assessment was included as an additional variable for the stage-related evaluation of symptom scores and CSF biomarkers. MMSE scores were included in models for the evaluation of *z*-values of CERAD items to correct for overall stage of cognitive impairment. The *z*-value of CERAD items represents the number of standard deviations distinct from cognitively normal control population, adjusted for age, sex, and education. Due to the study’s exploratory nature, no correction for multiple testing was done.

## Results

### Demographic data

The cohort included 228 patients (rpAD: *n* = 67; non rpAD: *n* = 161), with 29% experiencing a rapidly progressive course. Education years and sex showed similar distribution patterns in both groups and were not statistically significant. Patients in the rpAD group were on average 6 years younger than patients in the non-rpAD group (the median age of onset in the rpAD group was 68.4 (IQR = 11.94) years and 72.6 (IQR = 13.15) years in the non-rpAD group). Median total disease duration before baseline was 14 months (IQR = 21.7) in rpAD and 28 (IQR = 27.4) in non-rpAD (*p* < 0.001). The data and results of statistical comparisons are shown in Table 1.

**Table 1:** Demographic data

	**Overall cohort**	**rpAD**	**Non-rpAD**	***P*-value**
**Number**	**228**	**67**	**161**	
**Sex**: male	107 (47%)	29 (43%)	78 (48%)	0.560
**Sex**: female	121 (53%)	38 (57%)	83 (52%)	
**Age at onset (years):** median (IQR)	71.8 (13.2)	68.4 (11.9)	72.6 (13.2)	0.121
**Age at baseline (years):** median (IQR)	73.8 (14.7)	69.5 (13.1)	75.2 (13.5)	0.018*
**Duration at baseline (months):** median (IQR)	24.7 (25.1)	14.5 (21.7)	27.9 (27.4)	< 0.001*
**Education (years):** < 8/8/10/12/ > 12	3%/44%/28% / 5%/20%	2%/49%/30% / 6%/14%	4%/42%/26% / 5%/23%	0.383
**MMSE score at baseline**: median (IQR)	24 (7)	19 (11)	25 (5)	< 0.001*

*Abbreviations*: *rpAD* rapidly progressive Alzheimer’s disease, *IQR* interquartile range, *MMSE* Mini-Mental State Examination

^*^p-values below significance threshold of 0.05

### Clinical presentation

When comparing symptom complexes between rpAD and non-rpAD, affective symptoms were most frequent among psychiatric/behavioral symptoms (rpAD = 76%; non-rpAD = 64%), followed by sleep disturbance (rpAD = 33%; non-rpAD = 24%), and psychotic symptoms (rpAD = 12%; non-rpAD = 9%). In the focal neurological symptoms complex, extrapyramidal symptoms (rpAD = 33%; non-rpAD = 22%) were most common, followed by ataxia (rpAD = 13%; non-rpAD = 8%) and pyramidal symptoms (rpAD = 7%; non-rpAD = 1%). Although the frequency of all psychiatric/behavioral as well as neurological symptom groups was apparently higher among rpAD patients, no significant difference between the patient groups could be seen after correction for confounders (Table 2, details on regression analyzes in Additional file 1 B).

**Table 2:** Clinical symptom complexes

	**Overall cohort** *n* (yes/no), (%)	**rpAD** *n* (yes/no), (%)	**Non-rpAD** *n* (yes/no), (%)	***P*-value** (after MLRA)
**Behavioral and psychiatric symptoms**				
Affective symptoms	142/69 (67%)	47/15 (76%)	96/54 (64%)	0.109 (0.200)
Psychotic symptoms	21/194 (10%)	7/53 (12%)	14/141 (9%)	0.610 (0.381)
Sleep disturbances	57/157 (27%)	20/41 (33%)	37/116 (24%)	0.231 (0.323)
**Focal neurological signs**				
Pyramidal signs	5/199 (2%)	4/57 (7%)	1/141 (1%)	0.029 (0.083)
Extrapyramidal signs	53/160 (25%)	20/41 (33%)	33/119 (22%)	0.114 (0.056)
Ataxia	20/192 (9%)	8/52 (13%)	12/140 (8%)	0.295 (0.379)

Affective symptoms: apathy/drive reduction, anxiety, depression, euphoria, labile affect. Psychotic symptoms: delusion, hallucinations acoustic/visual/other. Sleep disturbances: sleep maintenance insomnia, sleep onset insomnia, day night reversal. Pyramidal signs: myoclonus, Babinski’s sign. Extrapyramidal signs: rigidity, resting tremor, hypokinesia. Ataxia: gait ataxia, static ataxia, truncal ataxia, appendicular ataxia

*Abbreviations*: *rpAD* rapidly progressive Alzheimer’s disease, *MLRA* multiple logistic regression analysis

Patients in the rpAD group scored lower than patients in the AD group on both ADL questionnaires (*p* < 0.001 for both comparisons after correction for cofactors). In rpAD, median BADL score was 44 (IQR = 8) and IADL score was 5 (IQR: 5), whereas non-rpAD patients had a median of 46 of (IQR: 4) of BADL and 7 of IADL (IQR: 3) scores. On the UPDRS III, patients from the rpAD group scored higher (median: 11, IQR: 15.25) than patients in the non-rpAD group (median 4, IQR: 8) and were thus clinically more affected (*p* < 0.001 after correction for cofactors). Please see Table 3 (Additional file 1 C) for a summary of the related data.

**Table 3:** Comparative evaluations of clinical scores

**Clinical scores**^**a**^	**Overall cohort**	**rpAD**	**Non-rpAD**	***P*-value** (*p*-value MLRA)
**BADL**	46 (5)	44 (8)	46 (4)	0.001 (< 0.001)*
**IADL**	6 (4)	5 (5)	7 (3)	0.001 (< 0.001)*
**UPDRS III**	6 (10)	11 (15.25)	4 (8)	< 0.001 (< 0.001)*
**Depression scale**^**b**^ patients	2 (3)	2 (3,5)	2 (3)	0.447 (0.642)
**Depression scale**^**b**^ relatives	3 (3)	3 (2.75)	3 (3)	0.797 (0.847)
**CERAD plus test item**^**c**^ (*n*: rpAD/*n*: non-rpAD)				
Semantic fluency (40/56)	− 1.7 ± 1.0	− 2.14 ± 0.9	− 1.4 ± 1.0	0.008*
Boston Naming Test (41/56)	− 1.14 ± 1.4	− 1.2 ± 1.5	− 1.1 ± 1.4	0.726
Word list learning (38/56)	− 3.0 ± 1.6	− 3.6 ± 1.5	− 2.6 ± 1.5	0.007*
Word list recall (38/56)	− 2.5 ± 1.2	− 2.7 ± 1.2	− 2.3 ± 1.2	0.395
Word list intrusions (38/56)	− 1.0 ± 1.2	− 1.1 ± 1.2	− 0.8 ± 1.2	0.228
Word list savings (%)(35/56)	− 2.2 ± 1.8	− 2.3 ± 2.0	− 2.1 ± 1.8	0.806
Discriminability (%)(37/55)	− 2.4 ± 1.5	− 2.5 ± 1.6	− 2.3 ± 1.5	0.928
Figure drawings (41/56)	− 1.5 ± 1.8	− 1.4 ± 1.8	− 1.5 ± 1.9	0.201
Figure recalls (40/56)	− 2.7 ± 1.3	− 3.0 ± 1.4	− 2.7 ± 1.2	0.909
Figure savings (%) (40/55)	− 2.1 ± 1.3	− 2.2 ± 1.2	− 2.1 ± 1.3	0.804
Phonematic fluency (39/54)	− 0.8 ± 1.2	− 1.3 ± 1.1	− 0.5 ± 1.2	0.023*
Trail Making Test A (34/47)	− 1.7 ± 1.3	− 1.9 ± 1.3	− 1.4 ± 1.2	0.178
Trail Making Test B (9/37)	− 1.6 ± 1.1	− 2.2 ± 0.6	− 1.4 ± 1.2	0.151

*Abbreviations*: *rpAD* rapidly progressive Alzheimer’s disease, *BADL* Basic Activities of Daily Living, *IQR* interquartile range, *IADL* Instrumental Activities of Daily Living, *UPDRS III* Unified Parkinson’s Disease Rating Scale

^a^Clinical scores are displayed as median and inter quartile range. *P*-values were derived from Mann–Whitney *U* test and MLRA, multiple logistic regression analyses including disease duration and age

^b^Depression scale: 0 = not depressed; 10 = most severe level of depression. Scores are displayed as median and inter quartile range

^c^Logistic regression analyses were performed using z-values (adjusted for age, sex and education) of all CEARD test items. *Z*-values are displayed as mean and standard deviation. In each model, the patient’s MMSE score was included as confounding variable to adjust for overall cognitive disease stage

^*^p-values below significance threshold of 0.05

Patients in the rpAD group had a median MMSE score of 19 (IQR = 11) points at baseline. Patients in the non-AD group had 25 (IQR = 5) points, showing a statistically significant difference (*p* < 0.001). Thus, MMSE scores were included as confounding variable in regression models to investigate influence of groups on different CERAD items. Here, worse performance in semantic verbal fluency (*p* = 0.008), phonematic verbal fluency (*p* = 0,023), and “word list learning” (*p* = 0.007) were significantly associated with rpAD. Other CERAD subtests did not show significant association with group assignment (Table 3, Additional file 1 C).

### Cerebrospinal fluid biomarkers

**Fig. 1:**
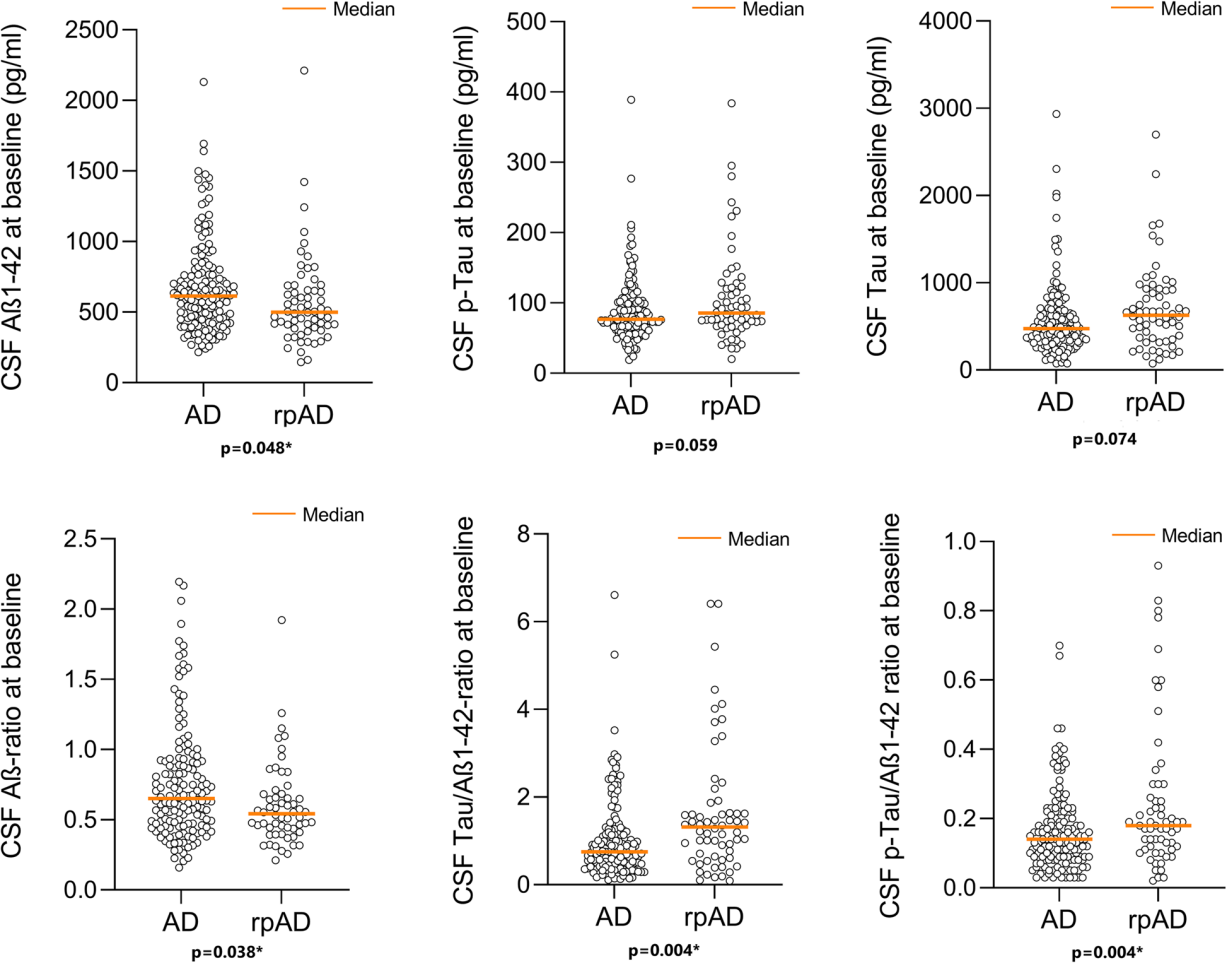
Cerebrospinal fluid biomarkers at baseline. Cerebrospinal fluid biomarker concentrations in diagnostic groups. Comparison of biomarker concentrations of diagnostic groups was performed with Mann–Whitney *U* tests. Results were corrected by multiple logistic regressions in a second step for the confounders “age at lumbar puncture” and “duration of disease at lumbar puncture.” *P*-values from regression models are indicated below dot columns. *p-values below significance threshold of 0.05. Bar indicates median. CSF, cerebrospinal fluid; rpAD, rapidly progressive Alzheimer’s disease; Aβ, amyloid beta

We compared concentrations of CSF were the amyloid markers (Aβ1-42 and Aβ-ratio) and Tau markers (Tau, p-Tau, and p-Tau/Tau ratio) between the two groups (Table 4, Fig. 1, Additional file 1 D).

**Table 4:** Alzheimer’s cerebrospinal fluid biomarkers at baseline

	**Overall cohort**	**rpAD**	**Non-rpAD**	***P*-value** (after MLRA)
**Tau (pg/ml)** median (IQR)	514 (387)	629 (562.3)	476 (338.5)	0.014* (0.074)
**P-Tau (pg/ml)** median (IQR)	79 (40)	85.5 (54.55)	77 (37)	0.090 (0.059)
**Tau-ratio (P-Tau/Tau)** median (IQR)	0.16 (0.09)	0.16 (0.1)	0.17 (0.08)	0.571 (0.336)
**Aβ1-42 (pg/ml)** median (IQR)	582 (343)	500 (274)	614 (338)	0.008* (0.048)*
**Aβ1-40**_**modified**_** (pg/ml)**^**a**^ median (IQR)	9415 (4152)	9145 (4035)	9459 (4218)	0.921 (0.339)
**Aß-ratio**_**modfied**_^**a**^ median (IQR)	0.62 (0.41)	0.54 (0.25)	0.65 (0.45)	0.008* (0.038)*
**Tau/Aβ1-42- Ratio** median (IQR)	0.9 (0.94)	1.32 (1.07)	0.75 (0.77)	< 0.001* (0.004)*
**P-Tau/Aβ1-42- Ratio** median (IQR)	0.15 (0.13)	0.18 (0.18)	0.14 (0.13)	0.007* (0.004)*

^a^Due to a change in laboratory methods the Aβ1-40 values had to be modified as described in the “Methods” section

*Abbreviations*: *rpAD* rapidly progressive Alzheimer’s disease, *MLRA* multiple logistic regression analysis, *IQR* interquartile range, *P-Tau* hyperphosphorylated Tau protein

^*^p-values below significance threshold of 0.05

The median level of CSF Aβ1-42 was significantly lower (*p* = 0.008, *p* = 0.048 after correction for confounders) in rpAD patients (500 pg/ml, IQR = 274) than in non-rpAD patients (614 pg/ml, IQR = 338). Consequently, the Aβ-ratio (42/40) was also lower in the rpAD (0.54, IQR = 0.23) than in the non-rpAD (0.65, IQR = 0.45) group (*p* < 0.001, *p* = 0.004 after correction for confounders). The median of Aβ1-40 was 9145 pg/ml (IQR = 4035) group rpAD and 9459 pg/ml (IQR = 4218) in the non-rpAD group, showing no statistically significant difference. The median CSF levels of total Tau (rpAD: 629 pg/ml, IQR = 562.3; non-rpAD: 476 pg/ml, IQR = 338.5) differed within the groups (*p* = 0.014), but after correction for age and disease duration, no statistical significance was observed. The medians of p-Tau (rpAD: 85.5 pg/ml, IQR = 54.55; non-rpAD: 77 pg/ml, IQR = 37) as well as Tau-ratio (rpAD: 0.16, IQR = 0.1; non-rpAD: 0.17, IQR = 0.08) did not differ significantly between the two groups. The Tau/Aβ1-42 ratio (rpAD, median: 1.32, IQR = 1.07; non-rpAD, median: 0.75, IQR = 0.77) and the p-Tau/Aβ1-42-ratio (rpAD, median: 0.18, IQR = 0.18; AD, median: 0.14, IQR = 0.13) were higher in the rpAD group, which turned out to be highly significant in exploratory comparisons (Tau/Aß1-42: *p* < 0.001; p-Tau/Aβ1-42: *p* = 0.007) as well as in corrected regression models (Tau/Aβ1-42: *p* = 0.004; p-Tau/Aβ1-42: *p* = 0.004).

Regarding the retrospective evaluation of other CSF biomarkers, no significant differences of t-PrP, YKL-40, and LCN2 could be observed in relatively small cohorts (Table 5). Only NfL showed significantly higher values in the rpAD group (*p* = 0.024), but due to the small samples size, MLRA correction could not be performed.

**Table 5:** Other cerebrospinal fluid biomarkers at baseline

	**Overall cohort**	**rpAD**	**Non-rpAD**	***p*-value**
**NfL (pg/ml)** median (IQR)	1878 (1203) *n* = 43	2393 (3023) *n* = 12	1699 (1229) *n* = 31	0.024*
**t-PrP (ng/ml)** median (IQR)	45.2 (28.53) *n* = 47	44.5 (20.05) *n* = 29	45.5 (34.2) *n* = 18	0.811
**YKL-40 (pg/ml)** median (IQR)	365.5 (88.8) *n* = 16	368 (195) *n* = 8	348 (95.5) *n* = 8	0.234
**LCN2 (pg/ml)** median (IQR)	800 (500) *n* = 19	880 (700) *n* = 7	780 (276) *n* = 12	0.853
**α-Synuclein (pg/ml)** median (IQR)	302 (349.8) *n* = 16	302 (426.3) *n* = 10	313.5 (277.3) *n* = 6	0.713

*Abbreviations*: *rpAD*, rapidly progressive Alzheimer’s disease; *NfL*, neurofilament light chain; glial fibrillary acidic protein, *GFAP*; t-PrP, total Prion protein; *YKL-40*, chitinase-3-like protein 1; *LCN2*, lipocalin2; *IQR*, interquartile range

^*^p-values below significance threshold of 0.05. All biomarkers were measured before [29–33] and retrospectively re-evaluated

### APOE genotype

**Fig. 2:**
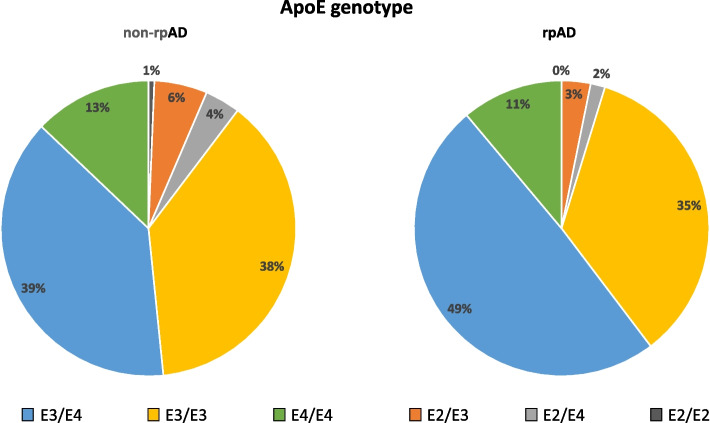
ApoE Genotype distribution at baseline. ApoE Genotype distribution in diagnostic groups. ApoE, apolipoprotein E; AD, Alzheimer’s disease; rpAD, rapidly progressive Alzheimer’s disease

The non-rpAD group’s most common APOE genotypes were E3/E4 (39%) and E3/E3 (38%), followed by the E4/E4 genotype with 13%; 37% had at least one APOE-4 allele. In the rpAD group, most patients had the E3/E4 genotype (49%), followed by E3/E3 (35%). Here, the prevalence of the ApoE4 allele was 40% (Fig. 2, Additional file 1 E). The proportion of E4 homozygous patients (11% in rpAD and 13% in non-rpAD) as well as the prevalence of the E4 allele carriers (62% of rpAD patients and 55% of non-rpAD patients) were similar in both groups but the rpAD group showed an apparently lower prevalence of the E2 allele carriers (5% vs. 10%). However, none of these differences were statistically significant (Additional file 1 F).

### Additional analyses in A + /T + patients

We performed the aforementioned analyses in a subgroup of AD patients that were both positive for AD-related amyloid and tau pathology according to the A/T/N system [21] to furtherly enhance the diagnostic accuracy and the homogeneity of the cohort. Decreased CSF Aβ-ratio or amyloid-PET positivity indicated A + ; T + was defined by elevated CSF pTau. Both biomarker categories were available and positive (A + /T +) in *n* = 117 AD patients (rpAD: *n* = 41; non-rpAD: *n* = 76). The frequency of A + /T + patients was higher in the rpAD group (*p* = 0.022). Further analyzes revealed that symptom complexes, clinical scores, CSF biomarkers, and APOE genotype distribution showed the similar tendencies compared to the whole cohort. UPDRS scores (*p* = 0.005) were still significantly higher and, BADL (*p* = 0.002) as well as IADL (*p* < 0.001) scores significantly lower in rpAD patients. Regarding biomarkers, the statistical significance was lost for Aβ1-42, Aβ-ratio, and the P-Tau/Aβ1-42 ratio but preserved for lower Tau/Aβ-42 ratio (*p* = 0.024). The results are summarized in Additional file 2 A-D.

## Discussion

Rapidly progressive cognitive decline in Alzheimer’s disease is a phenomenon that has been increasingly discussed in the literature in recent decades. In this study, we evaluated the clinical phenotype as well as CSF biomarkers in non-rpAD and rpAD in a comprehensive and comparative way. We identified several characteristics that differed significantly between the two types of the disease.

### Demographics and cohort characteristics

The proportion of rapid progression was 29%, which is in the upper range of prevalence in studies using this definition [4, 11, 12]. In our study, rpAD showed earlier disease onset (median age: 68.4 years) than non-rpAD patients (median age: 72.6), but significant differences between the groups were only observed regarding age at study baseline (rpAD: 69.5 years; non-rpAD: 75.2 years). Patients in both groups might develop the disease at a similar age but present to a physician earlier due to their extraordinary clinical course. Compared to the literature, our patients were younger at symptom onset and study entry, possibly due to different definitions of disease onset or chosen inclusion criteria. Other studies have hypothesized that age at symptom onset may differ between patients with non-rpAD and rpAD [10–13] and earlier disease onset might be associated with faster atrophy and increased neuritic plaques [10, 12]. However, some other studies did also not observe association between early onset and rpAD [18, 34, 35]. A recent review [14] concluded that the association between age at symptom onset and cognitive progression is not provable. This study did also not identify significant associations between rpAD and education level [18, 34] or sex [13, 18, 28, 34], in line with other reports. A potential explanation could be that the rapid cognitive decline in this disease subtype negates subtle effects from education level. However, some studies have described an association between higher education level and faster cognitive decline [10, 13].

### Clinical presentation and scores

We compared clinical symptoms based on symptom complexes (Table 2) to facilitate statistical analyzes. Most patients with Alzheimer’s will develop psychiatric symptoms and non-cognitive neurological symptoms [36], but their prevalence varies over disease stages, ranging from 12% in mild disease to > 90% in severe stages [37]. In our cohort, the groups did not differ significantly regarding occurrence of neuropsychiatric and focal neurological symptom complexes, which is in line with some studies that investigated association of cognitive decline and neuropsychiatric symptoms [35, 38] or focal neurological signs [12, 39] in AD. On the other hand, other studies identified neuropsychiatric symptoms and cognitive decline as potential prognostic of AD progression [14]. Likewise, some studies have shown a clear association between focal neurological symptoms and faster cognitive decline [13, 14, 34], as well as increased mortality and institutionalization [40]. In this regard, our results revealed that UPDRS III scores increased earlier in the disease course of patients with rpAD (rpAD median: 11, non-rpAD median: 4), primarily indicating earlier occurrence of extrapyramidal symptoms. Another study also examined the overall UPDRS III score in rpAD and found similar scores (median: 12) [41], which fits the hypothesis that extrapyramidal symptoms may be an indicators for faster functional and cognitive decline [10, 14]. Regarding functional impairment, the rpAD group scored significantly lower in both ADL scores, reflecting earlier functional impairment. Another study also reached similar conclusions [42]. However, a more recent review found no association between the rate of mental progression and ADL scores [13].

In terms of baseline cognitive level (MMSE score), the two groups differed significantly (median scores non-rpAD: 25; rpAD: 19). Similarly, other studies reported an increased risk of rapid progression in patients with lower baseline cognitive scores [12, 18, 43]. In this study, the MMSE was used as determinant of the global stage of cognitive functions and as a confounding variable to correct for disease stage in comparisons of the CERAD test items. We found significant association of worse performance in both verbal fluency tests as well as the initial word learning list with diagnosis of rpAD, independent of baseline MMSE scores (Table 3). This may be interpreted as an early occurrence of verbal fluency and immediate learning abilities in rpAD. Whereas early memory impairment is a key neuropsychological symptom in “classical” AD, semantic and verbal fluency reflect various cognitive domains, including executive functions and memory. It is intriguing that both tests are strongly related to language and speech functions, indicating early language impairment as a potential prognostic criterion in AD patients. The exploratory data warrants validation through specifically designed studies. So far, only few data on cognitive profiles in rpAD can be found in the literature but a study that investigated a general AD cohort found a significant association of better performance in both fluency tests with longer survival [44].

### Established cerebrospinal fluid biomarkers

CSF profiles may help to stratify patients for personalized future therapies [45] and allow interpretation of complex clinical trials [46] in AD. In this study, we found significant differences between groups in amyloid levels (Aβ1-42 and Aβ-ratio) and Tau/Amyloid ratios (Tau/Aβ1-42 and p-Tau/Aβ1-42), primarily indicating relatively lower CSF Aβ1-42 levels in rpAD, validating results from some other studies [4, 16]. Low Aβ1-42 levels and Aβ-ratios may be related to faster cognitive and functional deterioration [15, 16]. However, another study did not find a significant difference between the two groups in terms of amyloid markers [17].

We could not observe significant group differences regarding CSF Tau markers, similar to other studies on rpAD [16]. On the other hand, a high proportion of rpAD (8–16%) patients show extremely high CSF Tau (> 1250 pg/ml), when initially suspected CJD diagnosis was an inclusion criterion [3]. This might either be consequence of a selection bias (as Tau is a frequently used biomarker criterion for CJD) or associated with extremely rapid progression in those AD patients that had been under suspicion for CJD. The observations led to the hypothesis that high Tau levels occur mainly in diseases associated with intense neuronal degeneration (such as CJD). High levels indicate increased neuronal cell death [15], which suggests that the Tau levels in rpAD patients may be higher than in non-rpAD [3, 4]. In this study, medians of p-Tau (rpAD: 85.5 pg/ml; non-rpAD: 77 pg/ml) and the p-Tau/Tau ratio (rpAD, median: 0.16; AD, median: 0.17) showed no significant differences but a clear trend towards higher levels of p-Tau in rpAD. However, the data in the literature remains inconclusive as several studies described an association between faster disease progression and high Tau levels without a proportionally increased p-Tau value [15, 17]. An overview on further literature can be found in Additional file 1 G.

Biomarker ratios including Tau and Aβ are able to improve the diagnostic performance because they reflect the full pathological spectrum of AD [3, 16, 46]. We found significant differences between rpAD and non-rpAD patients regarding Tau/Aβ1-42 (*p* = 0.004) and p-Tau/Aβ1-42 (*p* = 0.004), which reflects the combination of relatively high Tau (or p-Tau, respectively) and relatively low Aβ1-42 levels in rpAD). Association of higher Tau/Aβ1-42 ratio with rapid disease course was also reported by other studies [15, 17, 45].

### Alternative cerebrospinal fluid biomarkers

In a retrospective analysis, we investigated emerging biomarker candidates for neuronal (or synaptic) damage, glial activation, and neuroinflammation in AD. No statistically significant differences of α-synuclein, YKL-40, t-PrP, and LCN2 between rpAD and non-rpAD could be observed, but due to the retrospective design, groups were rather small, and subtle differences may have been overlooked (Table 5). However, the t-PrP cohort was the biggest among these but medians were very similar between the groups (*p* = 0.811). Other studies investigated CSF t-PrP in “typical” AD “atypical” AD (AD with rapid cognitive and/or additional motor symptoms) and did not detected significant differences between these groups [47] or between AD and controls [48], respectively.

Regarding NfL, we observed higher concentrations in rpAD (*p* = 0.024), but due to the low group size (*n* = 12 rpAD patients), MLRA with consideration of age and disease duration could not be performed. Nonetheless, our results indicate that CSF NfL may be a promising marker for disease progression in patients with clinical diagnosis of AD. Other studies have shown that CSF NfL concentrations are potentially higher in atypical AD than in typical AD (no statistical significance) [49] and significantly higher in rapidly progressive than in normally progressive neurodegenerative dementia patients (not AD-specific) [50]. Interestingly, the latter study also showed higher CSF NfL in older AD patients than in younger ones, whereas our rpAD cases showed higher NfL despite younger age.

Alternative CSF and plasma or serum biomarkers may aid to the prediction of disease progression in AD and may contribute to the definition of rpAD. Several new candidates such as glial fibrillary acidic protein (GFAP) have been proposed. GFAP was shown to be elevated in preclinical stages and associated with disease stage as well as Aβ pathology [51]. However, researchers have to be cautious with retrospective analyzes: We measured CSF GFAP (SIMOA) in *n* = 62 AD patients (samples from 2008 to 2021) and found a strong correlation with sample age (lower levels in older samples, data not shown). Apparently, the measured concentrations were influenced by pre-analytical factors, which have to be taken into account in future investigations. We provide a list of promising “biomarkers of interest” in the supplements (Additional file 1 H).

### APOE genotypes

We could not find significant differences between the groups regarding APOE genotypes. Sixty percent of the patients with rpAD had at least one APOE4 allele and 54% in patients with non-rpAD. Regarding this, previous findings are inconsistent. Whereas some studies observed an influence of the APOE4-allele on disease progression in AD [13, 52] and association of increased amyloid deposition and faster atrophy, especially of the hippocampus [53], others failed to demonstrate an association between cognitive progression and APOE genotype [14, 17, 35]. Studies have even associated APOE4 presence with a reduced risk of rapid cognitive progression, describing a reduced frequency of APOE4 in rpAD cohorts [54, 55]. This discrepancy may be explained by APOE4 gene variations such as heterogeneity in single nucleotide polymorphisms (SNPs) [56], but it has also been hypothesized that alternative mechanisms gain the upper hand and negate the influence of APOE4 in the later course of the disease [54]. Another reason for discordant results may be the use of different rpAD definitions or the presence of different subtypes of rpAD. For example, an earlier investigation of our center’s cohort revealed low prevalence of APOE4 in rpAD [57]. At that time, most patients were included after initial suspicion of CJD, potentially representing an extremely aggressive subtype in the spectrum of rpAD. A literature overview on APOE genotypes and disease progression in AD can be found in Additional file 1 G.

### Study strengths and limitations

Many studies reported on rpAD cases with initial suspicion of CJD [2–4]. Other studies examined patient collectives from memory outpatient clinics or tertiary centers [11, 44, 58]. Here, we recruited participants with rpAD from both a memory outpatient clinic (*n* = 36) and the CJD Surveillance Centre (*n* = 31). A methodological advantage is the subsequent correction of significant results according to confounders. Also, the use of CSF ratios in the present study can be considered as a clear methodological advantage. The limitation includes the lack of genetic information from most of the patients. Although no patient had a suggestive family history (relatives with early onset AD), we cannot exclude the influence of unrevealed PSEN1, PSEN2, or APP mutations. Furthermore, groups with additional biomarker analyzes were relatively small. Especially regarding YKL-40, LCN2, and α-synuclein, subtle differences may not have become apparent from a statistical point of view. Another limitation is the missing of a final confirmation of the diagnosis and the analysis of relevant comorbidities by autopsy, such as Lewy bodies, one of the most common associated pathologies associated with a more pronounced and aggressive course in AD patients [35, 59]. Subgroup analyzes in A + /T + patients increased the probability of present AD-pathology and confirmed most of our results. However, some CSF biomarker differences could not be replicated. This has to be interpreted with caution because A and T positivity was defined by the same biomarkers that were investigated for group differences, which may cause a selection bias.

### Definitions and implications of rapidly progressive Alzheimer’s disease

The definition of rpAD has been a matter of discussions for many years and the MMSE has been used as a scale that reflects cognitive decline in AD in numerous studies (Additional file 3). Here, we validated that the cut-off at a loss of ≥ 6 MMSE points per year [4, 28] is associated with distinct clinical characteristics and biomarker profiles between rpAD and non-rpAD. We propose to employ this cut-off in future studies on fluid or imaging biomarkers and to consider the disease subtypes in clinical studies. Different clinicopathological characteristics may also be associated with different response to clinical intervention.

However, several points need clarification. Although being widely employed and well-validated, the MMSE is not the only test for global cognitive function. Despite exhaustive test batteries, other screening scores, such as the Montreal Cognitive Assessment (MoCA), should as well be considered in validation studies. Furthermore, cognitive decline is not the only measure for rapid disease progression. Alternative definition may include presence of atypical symptoms [47] or total disease duration [60]. Our data suggest different clinical and paraclinical characteristics when the MMSE cut-off is used, but these differences seem to be gradual. Extremely accelerated disease courses with survival time below 2 years may occur and should be investigated for concomitant pathologies and the potential presence of another distinct clinicopathological AD-subtype.

## Conclusions

In this study, we provide results from comparative analyses of clinical and CSF biomarker profiles in patients with “classical” non-rpAD and rpAD defined by a loss of ≥ 6 MMSE points per year. Patients with rpAD were younger at clinical admission showed worse performance in cognitive tests, earlier impairment of functional abilities, and occurrence of movement disturbances evaluated by the UPDRS III. This information, together with CSF Aβ1-42 values and distinct neuropsychological profiles, may allow identification of individuals with rapid disease progression. However, there are still open questions as our results are of exploratory nature. Future prospective studies may take these clinical characteristics into account when evaluating prognostic algorithms for AD. The study again illustrates the relevance of rpAD for everyday clinical practice and clinical research. The main goal should be to establish a recognized definition for rpAD including dynamic biomarker characteristics to enable a targeted study design and better comparability of the results in the future.

## Supplementary Information

**Additional file 1.** A. Calculation to ensure comparability of beta-amyloid 1-40 measurements. B. Results from logistic regression analysis of symptom complexes. C. Results from logistic regression analysis of clinical scores. D. Results from logistic regression analysis of CSF biomarkers. E. Allelic  combinations of APOE Genotypes. F. Presence of individual APOE alleles. G. Influence of biomarkers and APOE genotype on cognitive decline. H. Fluid biomarkers of interested for determination of Alzheimer’s Disease progression.

**Additional file 2.** A. Clinical symptom complexes in A+/T+ patients. B. Comparative evaluations of clinical scores in A+/T+ patients. C. Cerebrospinal fluid biomarkers at baseline in A+/T+ patients. D. Presence of individual APOE alleles in A+/T+ patients.

**Additional file 3.** Suggested criteria for discrimination or rapidly progressive and non-rapidly progressive Alzheimer’s Disease.

## Data Availability

The datasets used and analyzed during the current study are available from the corresponding author on reasonable request.

## Abbreviations

AD: Alzheimer’s disease
rpAD: Rapidly progressive Alzheimer’s disease
BPSD: Behavioral and Psychological Symptoms of Dementia
Aβ1-42: Amyloid-Beta 1–42
APOE: Apolipoprotein E gene
CJD: Creutzfeldt-Jakob disease
MMSE: Mini-Mental State Examination
ADL: Activity of Daily Living
UPDRSIII: Unified Parkinson’s Disease Rating Scale-III
CSF: Cerebrospinal fluid
p-Tau: Phosphorylated Tau protein
EPS: Extrapyramidal symptoms
