# Correction: Comparative evaluation of clinical and cerebrospinal fluid biomarker characteristics in rapidly and non‑rapidly progressive Alzheimer’s disease

**DOI:** 10.1186/s13195-023-01263-0

**Published:** 2023-06-22

**Authors:** Janne Marieke Herden, Peter Hermann, Isabel Schmidt, Kathrin Dittmar, Sezgi Canaslan, Luise Weglage, Sabine Nuhn, Corinna Volpers, Astrid Schlung, Stefan Goebel, Fabian Kück, Anna Villar‑Piqué, Christian Schmidt, Dirk Wedekind, Inga Zerr

**Affiliations:** 1 Department of Neurology, Clinical Dementia Center and National Reference Center for CJD Surveillance, University Medical Center, Robert‑Koch‑Straße 40, 37075 Göttingen, Germany; 2 German Center for Neurodegenerative Diseases (DZNE), Göttingen, Germany; 3 Department of Medical Statistics, University Medical Center Göttingen, Humboldtallee 32, 37073 Göttingen, Germany; 4 Neurologische Gemeinschaftspraxis Am Groner Tor, Göttingen, Germany; 5 Department of Psychiatry and Psychotherapy, University Medical Center, Von‑Siebold‑Straße 5, 37075 Göttingen, Germany

**Correction: Alzheimers Res Ther 15, 106 (2023)**

**https://doi.org/10.1186/s13195-023-01249-y**

Following publication of the original article [1], the authors identified an error in Fig. 2 (the color coding of APOE genotypes E2/2 and E3/4 is wrong). APOE2/E2 is displayed as most frequent genotype but in fact, it is the least frequent genotype. The correct figure is given below.

**Fig. 2:**
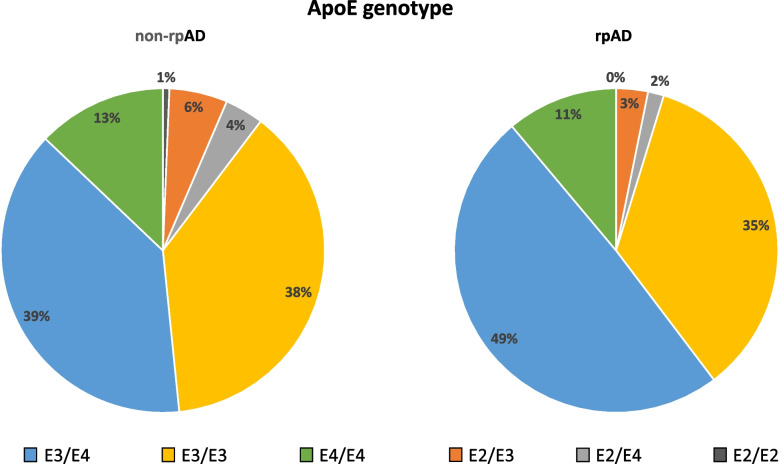
ApoE Genotype distribution at baseline. ApoE Genotype distribution in diagnostic groups. ApoE, apolipoprotein E; AD, Alzheimer’s disease; rpAD, rapidly progressive Alzheimer’s disease

The original article [1] has been corrected.
