# Supplementary material for: Comparative evaluation of clinical and cerebrospinal fluid biomarker characteristics in rapidly and non-rapidly progressive Alzheimer’s disease
Source: Alzheimers Res Ther. 2023 Jun 8;15:106. doi: 10.1186/s13195-023-01249-y (PMC10249304; doi:10.1186/s13195-023-01249-y)
Supplement: Supplementary file 1 — Additional file 1. A. Calculation to ensure comparability of beta-amyloid 1-40 measurements. B. Results from logistic regression analysis of symptom complexes. C. Results from logistic regression analysis of clinical scores. D. Results from logistic regression analysis of CSF biomarkers. E. Allelic combinations of APOE Genotypes. F. Presence of individual APOE alleles. G. Influence of biomarkers and APOE genotype on cognitive decline. H. Fluid biomarkers of interested for determination of Alzheimer’s Disease progression. [file 13195_2023_1249_MOESM1_ESM.pdf]

ADDITIONAL FILES

Additional File 1 A. Calculation to ensure comparability of beta-amyloid 1-40 measurements

In February 2010, there was a change of analytic conditions in the Göttingen neurochemistry laboratory for Beta-Aymloid 1-40. Values from before and after had to be adjusted to allow a comparison. For the calculation of Beta-Amyloid 1-40 values determined before February 2010, the following formula was used:

$$Aß1 - 40_{modified} = Aß1 - 40_{before} \times \frac{\bar{x}_1 + \bar{x}_2}{\bar{x}_1 \times 2}$$

For the calculation of Beta-Amyloid 1-40 values determined after February 2010, the following formula was used:

$$Aß1 - 40_{modified} = Aß1 - 40_{after} \times \frac{\bar{x}_1 + \bar{x}_2}{\bar{x}_2 \times 2}$$

$\bar{x}_1$ denotes the mean value of all Aß1-40 values before, $\bar{x}_2$ the mean value of all Beta-Amyloid 1-40 values after February 2010.

Additional File 1 B. Results from logistic regression analysis of symptom complexes

Variable	Non-rpAD/rpAD: n (n of positives)	Odds ratio	95% Confidence interval	P-value
Affective symptoms	150 (96)/ 62 (47)	0,63	0.31-1.26	0.200
Psychotic symptoms	155 (14)/ 60 (7)	0,64	0.24-1.83	0.381
Sleep disturbances	153 (37)/ 61 (20)	0,71	0.36-1.42	0.323
Pyramidal signs	142 (1)/ 61 (4)	0,13	0.01-1.07	0.083
Extrapyramidal signs	152 (33)/ 61 (20)	0,50	0.24-1.02	0.056
Ataxia	152 (12)/ 60 (8)	0.62	0.22-1.86	0.379

Affective symptoms: apathy/drive reduction, anxiety, depression, euphoria, labile affect. Psychotic symptoms: delusion, hallucinations acoustic/ visual/ other. Sleep disturbances: sleep maintenance insomnia, sleep onset insomnia, day night reversal. Pyramidal signs: myoclonus, Babinski's sign. Extrapyramidal signs: rigidity, resting tremor, hypokinesia. Ataxia: gait ataxia, static ataxia, truncal ataxia, appendicular ataxia. Abbreviations: rpAD, rapidly progressive Alzheimer's disease

Additional File 1 C. Results from logistic regression analysis of clinical scores

Variable	Non-rpAD/rpAD (n)	Odds ratio	95% Confidence interval	P-value
BADL	148/60	1.18	1.10-1.28	<0.001
IADL	149/62	1.45	1.26 -1.70	<0.001
UPDRS III	140/54	0.89	0.85-0.93	<0.001
Depression Scale patients	121/41	1.05	0.87-1.26	0.642
Depression Scale relatives	100/36	1.02	0.85-1.24	0.847
**CEARD test battery**				
Semantic fluency	56/40	0.51	0.31-0.84	0.008
Boston Naming Test	56/41	1.06	0.77-1.45	0.726
Word list learning	56/38	0.62	0.43-0.88	0.007
Word list recall	56/38	0.83	0.55-1.27	0.395
Word list intrusions	56/38	0.80	0.56-1.15	0.228
Word list savings	56/35	0.97	0.75-1.25	0.806
Discriminability	55/37	1.01	0.76-1.35	0.928
Figure drawings	56/41	1.18	0.92-1.52	0.201
Figure recalls	56/40	0.98	0.69-1.39	0.909
Figure savings	55/40	1.04	0.74-1.47	0.804
Phonematic fluency	54/39	0.59	0.38-0.93	0.023
Trail Making Test A	47/34	0.77	0.53-1.13	0.178
Trail Making Test B	37/9	0.48	0.17-1.31	0.151

Logistic regression analyses were perfomed using z-values (MMSE scores adjusted for age, sex and education) of all CEARD test items. In each model, the patient's MMSE score was included as confounding variable to adjust for overall cognitive disease stage.

Additional File 1 D. Results from logistic regression analysis of CSF biomarkers

Variable	Non-rpAD/rpAD (n)	Odds ratio	95% Confidence interval	P-value
Tau	151/60	1.00	1.00-1.00	0.074
P-Tau	152/59	1.00	0.99-1.00	0.059
Tau-ratio (P-Tau/Tau)	148/59	7.59	0.14-558.90	0.336
Aß1-42 (pg/ml)	151/60	1.00	1.00-1.00	0.048
Aß1-40$_{modified}$[a]	149/57	1.00	1.00-1.00	0.339
Aß-ratio$_{modfied}$[a]	149/57	2.93	1.13-8.73	0.038
Tau/Aß1-42- Ratio	147/60	0.67	0.50-0.87	0.004
P-Tau/Aß1-42- Ratio	151/59	0.05	0.01-0.37	0.004

[a]Due to a change in laboratory methods the Aß1-40 values had to be modified as described in the "Methods" section. Abbreviations: rpAD, rapidly progressive Alzheimer's disease; MLRA, Multiple Logistic Regression Analysis; IQR, interquartile range; P-Tau, hyperphosphorylated Tau protein**.**

Additional File 1 E. Allelic combinations of APOE Genotypes

**Allelic combinations**	**Overall cohort** Numbers n (%)	**rpAD** Numbers n (%)	**Non-rpAD** Numbers n (%)
	n = 218	n = 63	n = 155
E2/E2	1 (0%)	0 (0%)	1 (1%)
E2/E3	11 (5%)	2 (3%)	9 (6%)
E2/E4	7 (3%)	1 (2%)	6 (4%)
E3/E3	81 (37%)	22 (35%)	59 (38%)
E3/E4	91 (42%)	31 (49%)	60 (39%)
E4/E4	27 (12%)	7 (11%)	20 (13%)

Abbreviations: rpAD, rapidly progressive Alzheimer's disease

Additional File 1 F. Presence of individual APOE alleles

**Allele**	**rpAD** yes/no (% yes)	**Non-rpAD** yes/no (% yes)	***P*-value** (*Fisher's exact test*)
E2	3 /60 (5%)	16/ 139 (10%)	0.289
E3	55/8 (87%)	128/27 (83%)	0.425
E4	39/24 (62%)	86/69 (55%)	0.451

Abbreviations: rpAD, rapidly progressive Alzheimer's disease

Additional File 1 G. Influence of biomarkers and APOE genotype on cognitive decline

Reference	Cohort Size	Study design/ definition of AD	Significant outcomes
**CSF biomarkers**			
[1]	27	Autopsy confirmed rpAD cases/suspicion of CJD; disease; duration ≤2 years	-t-tau, p-tau, and 14-3-3 higher in rpAD than in typical AD patients
[2]	151	Patients with AD from a memory clinic	-low CSF p-tau-181/tau ratio and Aβ42, high tau and tau/Aβ42-ratio were associated with rapid decline
[3]	1791	Patients with AD, other dementias, and healthy controls	-Aβ1–42/Aβ1–40 CSF ratio was associated with clinical progression
[4]	312	Mild AD patients<br>rpAD: MMSE score loss ≥4 points within 6 months	-no differences (rpAD/non-rpAD) of CSF tau, p-tau, amyloid-β<br>- lower p-tau/tau ratio in rpAD
[5]	151	Outpatients with clinical diagnosis of AD	- extreme CSF biomarkers levels were associated faster cognitive decline and a higher mortality
**APOE**			
[6]	-	Systemic review and meta-analysis	-increased risk of rpAD in AD: APOE4
[7]	-	Literature review	-unclear association of APOE4 and rate of cognitive decline
[4]	312	Mild AD patients; rpAD: MMSE score loss ≥4 points within 6 months	-no differences between non- and rpAD across APOE ε4/ε4, APOE ε3/ε4, and APOE ε2/ε4 genotype distribution
[8]	290	Clinical diagnosis of AD<br>rpAD: MMSE score loss ≥3 points within 12 months	- No significant difference between groups regarding APOE4 alleles
[9]	218	Clinical diagnosis of AD	- APOE genotype predicts rate of cognitive decline, dose-response relationship with APOE4-allele<br>- APOE2-allele was s protective
[10]	414 / 156	Clinical diagnosis of AD, from several longitudinal studies	- presence of at least one APOE4 allele was associated with faster cognitive decline
[11]	189	Clinical diagnosis of AD from a longitudinal cohort study	-Presence of two APOE4 alleles was associated with a slower rate of clinical progression
[12]	65	Clinical diagnosis of AD from a memory clinic	- Absence of APOE4 alleles was associated with faster whole-brain atrophy rate

Abbreviations: AD, Alzheimer's disease; rpAD, rapidly progressive Alzheimer's disease; CJD, Creutzfeldt-Jakob disease; MMSE, Mini Mental Status Examination; CSF, cerebrospinal fluid; ChEI, cholinesterase inhibitor; Aβ, Beta-amyloid; APOE, Apolipoprotein E Gene;

Additional File 1 E. Fluid biomarkers of interest for determination of Alzheimer's Disease progression

Biomarker	
**Tau**	
pTau217	• Plasma pTau217 is associated with slopes of longitudinal cognitive measures [13]
**Synaptic proteins**	
Neurogranin	• CSF Neurogranin is elevated in AD and predicts conversion of MCI to AD [14]
SNAP-25	• CSF SNAP-25 is an early and potentially dynamic biomarker for AD [15]
β-Synuclein	• CSF and serum β-Synuclein are dynamic markers for AD and reflect synaptic damage, atrophy, and Aβ-pathology [16, 17]
**Astroglial proteins**	
GFAP	• Plasma GFAP tracks astrogliosis and Aβ-pathology in AD [18]
YKL-40	• YKL-40 may induce neurodegeneration via neuroinflammation [19]
LCN2	• Plasma Lipoclain-2 is lower in rpAD than in non-rpAD [20]
**Microglial proteins**	
sTREM1	• Dynamic up-regulation of CSF sTREM1 in AD [21]
sTREM2	• High CSF [22] and plasma [23] sTREM2 is associated with a faster cognitive decline
**Others**	
micro RNAs	• Dysregulted miRNAs are associated with pathophysiological mechanisms of disease progression [24]
mitochondrial DNA	• Decreased CSF levels are associated with slow disease progression [25]
MIF (and other cytokines)	• CSF MIF is increased in AD and correlates with pTau [21]
SFPQ	• Down-regulation in post-mortem rpAD brains compared to non-rpAD and potential modificator of Tau-pathology [26]
MBP	• Abscence of MBD is assoicated with amyloidogenic APP-processinig [27]
MFG-E8	• Down-regulation in CSF as potential marker of Amyloid-Angiopathy [28]

This table provides a list of some potential biomarkers for rapidly-progressive Alzheimer's disease (rpAD). This is an overview and we do not claim to be exhaustive regarding the candidates and the references. In widely investigated markers, meta-analyzes were favoured over original research references.
AD: Alzheimer's disease; SNAP-25: Synaptosomal Associated Protein-25; MIF: Migration inhibitory factor SFPQ: Splicing Factor Proline And Glutamine Rich; MBP: Myelin Basic Protein; MFG-E8: Milk fat globule-EGF factor 8 protein.

Bibliography of Additional file 1

1. Abu-Rumeileh S, Capellari S, Parchi P. Rapidly progressive Alzheimer's Disease: Contributions to clinical-pathological definition and diagnosis. J Alzheimers Dis. IOS Press; 2018;63:887–97.

2. Kester MI, van der Vlies AE, Blankenstein MA, Pijnenburg YAL, van Elk EJ, Scheltens P, et al. CSF biomarkers predict rate of cognitive decline in Alzheimer disease. Neurology. Wolters Kluwer Health, Inc. on behalf of the American Academy of Neurology; 2009;73:1353–8.

3. Delaby C, Estellés T, Zhu N, Arranz J, Barroeta I, Carmona-Iragui M, et al. The Aβ1–42/Aβ1–40 ratio in CSF is more strongly associated to tau markers and clinical progression than Aβ1–42 alone. Alzheimers Res Ther. 2022;14:20.

4. Ba M, Li X, Ng KP, Pascoal TA, Mathotaarachchi S, Rosa-Neto P, et al. The prevalence and biomarkers' characteristic of rapidly progressive Alzheimer's disease from the Alzheimer's Disease Neuroimaging Initiative database. Alzheimers Dement (N Y). 2017;3:107–13.

5. Wallin ÅK, Blennow K, Zetterberg H, Londos E, Minthon L, Hansson O. CSF biomarkers predict a more malignant outcome in Alzheimer disease. Neurology. Wolters Kluwer Health, Inc. on behalf of the American Academy of Neurology; 2010;74:1531–7.

6. Song YN, Wang P, Xu W, Li JQ, Cao XP, Yu JT, et al. Risk factors of rapid cognitive decline in Alzheimer's Disease and mild cognitive impairment: A systematic review and meta-analysis. Montero-Odasso M, editor. J Alzheimers Dis. 2018;66:497–515.

7. Loeffler DA. Modifiable, non-modifiable, and clinical factors associated with progression of Alzheimer's Disease. J Alzheimers Dis. IOS Press; 2021;80:1–27.

8. Nance C, Ritter A, Miller JB, Lapin B, Banks SJ. The pathology of rapid cognitive decline in clinically diagnosed Alzheimer's Disease. J Alzheimers Dis. IOS Press; 2019;70:983–93.

9. Martins C, Oulhaj A, de Jager CA, Williams JH. APOE alleles predict the rate of cognitive decline in Alzheimer disease: A nonlinear model. Neurology. Wolters Kluwer Health, Inc. on behalf of the American Academy of Neurology; 2005;65:1888–93.

10. Cosentino S, Scarmeas N, Helzner E, Glymour MM, Brandt J, Albert M, et al. APOE ε4 allele predicts faster cognitive decline in mild Alzheimer disease. Neurology. Wolters Kluwer Health, Inc. on behalf of the American Academy of Neurology; 2008;70:1842–9.

11. Hoyt BD, Massman PJ, Schatschneider C, Cooke N, Doody RS. Individual growth curve analysis of APOE ε4–associated cognitive decline in Alzheimer's Disease. Arch Neurol. 2005;62:454–9.

12. Sluimer JD, Vrenken H, Blankenstein MA, Fox NC, Scheltens P, Barkhof F, et al. Whole-brain atrophy rate in Alzheimer disease: Identifying fast progressors. Neurology. Wolters Kluwer Health, Inc. on behalf of the American Academy of Neurology; 2008;70:1836–41.

13. Mattsson-Carlgren N, Salvadó G, Ashton NJ, Tideman P, Stomrud E, Zetterberg H, Ossenkoppele R, Betthauser TJ, Cody KA, Jonaitis EM, Langhough R, Palmqvist S, Blennow K, Janelidze S, Johnson SC, Hansson O. Prediction of Longitudinal Cognitive

Decline in Preclinical Alzheimer Disease Using Plasma Biomarkers. JAMA Neurol. 2023;80:360−369.

14. Mavroudis IA, Petridis F, Chatzikonstantinou S, Kazis D. A meta-analysis on CSF neurogranin levels for the diagnosis of Alzheimer's disease and mild cognitive impairment. Aging Clin Exp Res. 2020;32:1639−1646.

15. Liu Q, Liu H, Zhang S, Yang Q, Shen L, Jiao B. Cerebrospinal Fluid Synaptosomal-Associated Protein 25 Levels in Patients with Alzheimer's Disease: A Meta-Analysis. J Alzheimers Dis. 2022;89:121−132.

16. Barba L, Abu Rumeileh S, Bellomo G, Paolini Paoletti F, Halbgebauer S, Oeckl P, Steinacker P, Massa F, Gaetani L, Parnetti L, Otto M. Cerebrospinal fluid β-synuclein as a synaptic biomarker for preclinical Alzheimer's disease. J Neurol Neurosurg Psychiatry. 2023;94:83−86.

17. Oeckl P, Anderl-Straub S, Danek A, Diehl-Schmid J, Fassbender K, Fliessbach K, et al. Relationship of serum beta-synuclein with blood biomarkers and brain atrophy. Alzheimers Dement. 2023;19:1358−1371.

18. Benedet AL, Milà-Alomà M, Vrillon A, Ashton NJ, Pascoal TA, Lussier F, et al. Differences Between Plasma and Cerebrospinal Fluid Glial Fibrillary Acidic Protein Levels Across the Alzheimer Disease Continuum. JAMA Neurol. 2021;78:1471–1483.

19. Connolly K, Lehoux M, O'Rourke R, Assetta B, Erdemir GA, Elias JA, Lee CG, Huang YA. Potential role of chitinase-3-like protein 1 (CHI3L1/YKL-40) in neurodegeneration and Alzheimer's disease. Alzheimers Dement. 2023;19:9−24.

20. Hermann P, Villar-Piqué A, Schmitz M, Schmidt C, Varges D, Goebel S, et al. Plasma Lipocalin 2 in Alzheimer's disease: potential utility in the differential diagnosis and relationship with other biomarkers. Alzheimers Res Ther. 2022 Jan 13;14:9.

21. Hok-A-Hin YS, Del Campo M, Boiten WA, Stoops E, Vanhooren M, Lemstra AW, et al. Neuroinflammatory CSF biomarkers MIF, sTREM1, and sTREM2 show dynamic expression profiles in Alzheimer's disease. J Neuroinflammation. 2023;20:107.

22. Ewers M, Franzmeier N, Suárez-Calvet M, Morenas-Rodriguez E, Caballero MAA, Kleinberger G, et al. Increased soluble TREM2 in cerebrospinal fluid is associated with reduced cognitive and clinical decline in Alzheimer’s disease. Sci Transl Med. 2019;11:eaav6221.

23. Zhao A, Jiao Y, Ye G, Kang W, Tan L, Li Y, et al. Soluble TREM2 levels associate with conversion from mild cognitive impairment to Alzheimer's disease. J Clin Invest. 2022;132:e158708.

24. Arora T, Prashar V, Singh R, Barwal TS, Changotra H, Sharma A, Parkash J. Dysregulated miRNAs in Progression and Pathogenesis of Alzheimer's Disease. Mol Neurobiol. 2022;59:6107−6124.

25. Podlesniy P, Llorens F, Puigròs M, Serra N, Sepúlveda-Falla D, Schmidt C, et al. Cerebrospinal Fluid Mitochondrial DNA in Rapid and Slow Progressive Forms of Alzheimer's Disease. Int J Mol Sci. 2020;21:6298.

26. Younas N, Zafar S, Shafiq M, Noor A, Siegert A, Arora AS, et al. SFPQ and Tau: critical factors contributing to rapid progression of Alzheimer's disease. Acta Neuropathol. 2020;140:317−339.

27. Seiwa C, Sugiyama I, Sugawa M, Murase H, Kudoh C, Asou H. The Absence of Myelin Basic Protein Reduces Non-Amyloidogenic Processing of Amyloid Precursor Protein. Curr Alzheimer Res. 2021;18:326−334.

28. Marazuela P, Solé M, Bonaterra-Pastra A, Pizarro J, Camacho J, Martínez-Sáez E, et al. MFG-E8 (LACTADHERIN): a novel marker associated with cerebral amyloid angiopathy. Acta Neuropathol Commun. 2021;9:154.
