# Supplementary material for: Comparative evaluation of clinical and cerebrospinal fluid biomarker characteristics in rapidly and non-rapidly progressive Alzheimer’s disease
Source: Alzheimers Res Ther. 2023 Jun 8;15:106. doi: 10.1186/s13195-023-01249-y (PMC10249304; doi:10.1186/s13195-023-01249-y)
Supplement: Supplementary file 2 — Additional file 2. A. Clinical symptom complexes in A+/T+ patients. B. Comparative evaluations of clinical scores in A+/T+ patients. C. Cerebrospinal fluid biomarkers at baseline in A+/T+ patients. D. Presence of individual APOE alleles in A+/T+ patients. [file 13195_2023_1249_MOESM2_ESM.pdf]

ADDITIONAL FILES

Additional File 2 A. Clinical symptom complexes in A+/T+ patients

	**Overall cohort** n (yes/no), (%)	**rpAD** n (yes/no), (%)	**Non-rpAD** n (yes/no), (%)	***P*-value** (after MLRA)
		**Behavioural and psychiatric symptoms**		
Affective symptoms	68/38 (64%)	27/12 (69%)	41/26 (61%)	0.529
Psychotic symptoms	10/98 (9%)	4/33 (11%)	6/65 (8%)	0.733
Sleep disturbances	25/82 (23%)	11/26 (30%)	14/56 (20%)	0.337
		**Focal neurological signs**		
Pyramidal signs	2/101 (2%)	2/35 (5%)	0/66 (0%)	0.127
Extrapyramidal signs	18/88 (17%)	9/28 (24%)	9/60 (13%)	0.177
Ataxia	7/99 (7%)	4/34 (11%)	3/65 (4%)	0.247

Affective symptoms: apathy/drive reduction, anxiety, depression, euphoria, labile affect. Psychotic symptoms: delusion, hallucinations acoustic/ visual/ other. Sleep disturbances: sleep maintenance insomnia, sleep onset insomnia, day night reversal. Pyramidal signs: myoclonus, Babinski's sign. Extrapyramidal signs: rigidity, resting tremor, hypokinesia. Ataxia: gait ataxia, static ataxia, truncal ataxia, appendicular ataxia. Abbreviations: rpAD, rapidly progressive Alzheimer's disease; MLRA, Multiple Logistic Regression Analysis.

Additional File 2 B. Comparative evaluations of clinical scores in A+/T+ patients

**Clinical scores[a]**	**Overall cohort**	**rpAD**	**Non-rpAD**	***P*-value** (*p*-value MLRA)
**BADL**	46 (4)	45 (8.25)	47 (3)	0.003* (0.002)*
**IADL**	7 (3.5)	6 (5)	7 (2.75)	0.001* (<0.001)*
**UPDRS III**	6 (9)	10 (11.25)	4 (7.75)	<0.001* (0.005)*
**Depression Scale[b]** patients	2 (3)	1 (3)	2 (4)	0.092
**Depression Scale[b]** relatives	3 (4)	3 (3)	3 (3.75)	0.688

Abbreviations: rpAD, rapidly progressive Alzheimer's disease;; BADL, Basic Activities of Daily Living; IQR, interquartile range; IADL, Instrumental Activities of Daily Living; UPDRS III, Unified Parkinson's Disease Rating Scale. [a]Clinical scores are displayed as median and inter quartile range. *P*-values were derived from Mann-Whtiney-U Test and MLRA, Multiple Logistic Regression Analyses inlcuding disease duration, age, and sex. [b]Depression Scale: 0= not depressed; 10= most severe level of depression. Scores are displayed as median and inter quartile range.

Additional File 2 C. Cerebrospinal fluid biomarkers at baseline in A+/T+ patients

	Overall cohort	rpAD	Non-rpAD	***P*-value** (after MLRA)
**Tau (pg/ml)**, median (IQR)	632 (461)	703 (400)	570 (485)	0.007* (0.074)
**P-Tau (pg/ml),** median (IQR)	98 (50.55)	108 (67.30)	94.5 (44.25)	0.110
**Tau-ratio (P-Tau/Tau)**, median (IQR)	0.164 (0.09)	0.15 (0.08)	0.18 (0.10)	0.190
**Aß1-42 (pg/ml)**, median (IQR)	480 (244.5)	468 (206.5)	485.5 (253.5)	0.566
**Aß1-40$_{modified}$ (pg/ml)[a],** median (IQR)	9242 (4660)	8839 (4911)	9684 (4395)	0.338
**Aß-ratio$_{modfied}$[a],** median (IQR)	0.54 (0.27)	0.54 (0.30)	0.55 (0.24)	0.900
**Tau/Aß1-42- Ratio**, median (IQR)	1.275 (1.35)	1.47 (1.82)	1.14 (1.42)	0.011* (0.024)*
**P-Tau/Aß1-42- Ratio**, median (IQR)	0.2 (0.14)	0.21 (0.23)	0.19 (0.12)	0.112

[a]Due to a change in laboratory methods the Aß1-40 values had to be modified as described in the “Methods” section. Abbreviations: rpAD, rapidly progressive Alzheimer’s disease; MLRA, Multiple Logistic Regression Analysis; IQR, interquartile range; P-Tau, hyperphosphorylated Tau protein. *p-values below significance threshold of 0.05.

Additional File 2 D. Presence of individual APOE alleles in A+/T+ patients

**Allele**	**rpAD** yes/no (% yes)	**Non-rpAD** yes/no (% yes)	***P*-value** (*Fisher’s exact test*)
E2	3/36 (4%)	6/67 (4%)	>0.999
E3	33/6 (42%)	54/19 (37%)	0.239
E4	23/16 (29%)	41/32 (28%)	0.843

Abbreviations: rpAD, rapidly progressive Alzheimer’s disease
