# Supplementary material for: Comparative evaluation of clinical and cerebrospinal fluid biomarker characteristics in rapidly and non-rapidly progressive Alzheimer’s disease
Source: Alzheimers Res Ther. 2023 Jun 8;15:106. doi: 10.1186/s13195-023-01249-y (PMC10249304; doi:10.1186/s13195-023-01249-y)
Supplement: Supplementary file 3 — Additional file 3. Suggested criteria for discrimination or rapidly progressive and non-rapidly progressive Alzheimer’s Disease. [file 13195_2023_1249_MOESM3_ESM.pdf]

ADDITIONAL FILES

Additional file 3. Suggested criteria for discrimination or rapidly-progressive and non-rapidly progressive Alzheimer's Disease

Reference	Definition of rpAD	Additional diagnostic criteria
**O'Hara et al. 2002 [1]**<br>**Carcaillon et al. 2007 [2]**	Loss of ≥ 3 points on the MMSE score per year	Clinical diagnosis of Alzheimer's disease
**Sona et al. 2012 [3]**	Loss of ≥ 6 points on the MMSE score in 18 months	Clinical diagnosis of Alzheimer's disease
**Doody et al. 2001 [4]**	Loss of ≥ 5 points on the MMSE score per year	Clinical diagnosis of Alzheimer's disease
**Dumont et al. 2003 [5]**<br>**Soto M et al. 2008 [6]**<br>**Schmidt et al. 2011 [7]**	Loss of ≥ 6 points on the MMSE score per year (or ≥ 3 in 6 months)	Clinical diagnosis of Alzheimer's disease
**Dumont et al. 2005 [8]**<br>**Ba et al. 2017 [9]**	Loss of ≥ 4 points on the MMSE score in 6 months	Clinical diagnosis of Alzheimer's disease
**Josephs et al. 2009* [10]**	Total disease duration (onset to death): < 4 years	Neuropathological diagnosis of neurodegenerative dementia

AD: Alzheimer-Demenz; MMST: Mini Mental Status Test; PRNP: Prion Protein

*No differentiation of AD and other neurodegenerative dementias

Bibliography of Additional file 3

1. O'Hara R, Thompson JM, Kraemer HC, Fenn C, Taylor JL, Ross L, Yesavage JA, Bailey AM, Tinklenberg JR. Which Alzheimer patients are at risk for rapid cognitive decline? J Geriatr Psychiatry Neurol. 2002;15:233−238.
2. Carcaillon L, Pérès K, Péré JJ, Helmer C, Orgogozo JM, Dartigues JF. Fast cognitive decline at the time of dementia diagnosis: a major prognostic factor for survival in the community. Dement Geriatr Cogn Disord. 2007;23:439−445.
3. Sona A, Zhang P, Ames D, Bush AI, Lautenschlager NT, Martins RN, et al. Predictors of rapid cognitive decline in Alzheimer's disease: results from the Australian imaging, biomarkers and lifestyle (AIBL) study of ageing. Int Psychogeriatr. 2012;24:197−204.
4. Doody RS, Massman P, Dunn JK. A method for estimating progression rates in Alzheimer disease. Arch Neurol. 2001;58:449−454
5. Dumont C, Gillette-Guyonnet S, Andrieu S, Cantet C, Ousset PJ, Vellas B. Baisse rapide du Mini Mental State Examination: étude REAL.FR [Rapid loss of the Mini Mental State Examination: REAL.FR study]. Rev Med Interne. 2003;24 Suppl 3:345s–350s. French.
6. Soto M, Andrieu S, Arbus C, Ceccaldi M, Couratier P, Dantoine T, et al. Rapid cognitive decline in Alzheimer’s disease. Consensus paper. J Nutr Health Aging. 2008;12:703–13.
7. Schmidt C, Wolff M, Weitz M, Bartlau T, Korth C, Zerr I. Rapidly progressive Alzheimer Disease. Arch Neurol. 2011;68:1124–30.
8. Dumont C, Voisin T, Nourhashemi F, Andrieu S, Koning M, Vellas B. Predictive factors for rapid loss on the mini-mental state examination in Alzheimer's disease. J Nutr Health Aging. 2005;9:163–167.
9. Ba M, Li X, Ng KP, Pascoal TA, Mathotaarachchi S, Rosa-Neto P, et al. The prevalence and biomarkers’ characteristic of rapidly progressive Alzheimer’s disease from the Alzheimer’s Disease Neuroimaging Initiative database. Alzheimers Dement (N Y). 2017;3:107–13.
10. Josephs KA, Ahlskog JE, Parisi JE, Boeve BF, Crum BA, Giannini C, et al. Rapidly progressive neurodegenerative dementias. Arch Neurol. 2009;66:201–207.
